# Efficacy of Immunotherapy Versus Chemotherapy in Advanced Pleural Mesothelioma: A Turkish Oncology Group (TOG) Study

**DOI:** 10.3390/medicina61040638

**Published:** 2025-03-31

**Authors:** Ziya Kalkan, Senar Ebinc, Murat Arcagok, Ahmet Bilici, Ozcan Yildiz, Saadettin Kilickap, Deniz Can Guven, Ali Murat Tatli, Ahmet Taner Sumbul, Nil Molinas Mandel, Akin Ozturk, Murat Bardakci, Serdar Karakaya, Muhammet Ali Kaplan

**Affiliations:** 1 Department of Medical Oncology, Mardin Training and Research Hospital, Mardin 47100, Türkiye; 2 Department of Medical Oncology, Van Yuzuncu Yil University Faculty of Medicine, Van 65100, Türkiye; 3 Department of Medical Oncology, Dicle University Faculty of Medicine, Diyarbakir 21100, Türkiye; 4 Department of Medical Oncology, Faculty of Medicine, Medipol University, Istanbul 34000, Türkiye; 5 Department of Medical Oncology, Istinye University Faculty of Medicine, Istanbul 34000, Türkiye; 6 Department of Medical Oncology, Hacettepe University Cancer Institute, Ankara 06000, Türkiye; 7 Department of Medical Oncology, Akdeniz University Faculty of Medicine, Antalya 07000, Türkiye; 8 Department of Medical Oncology, Baskent University Adana Dr. Turgut Noyan Application and Research Center, Adana 01100, Türkiye; 9 Department of Medical Oncology, American Hospital, Istanbul 34000, Türkiye; 10 Department of Medical Oncology, Sureyyapasa Chest Diseases and Chest Surgery Training and Research Hospital, Istanbul 34000, Türkiye; 11 Department of Medical Oncology, Gazi Yasargil Training and Research Hospital, Diyarbakir 21100, Türkiye; 12 Department of Medical Oncology, Ankara Ataturk Sanatoryum Training and Research Hospital, Ankara 06000, Türkiye

**Keywords:** pleural mesothelioma, immunotherapy, real-world data

## Abstract

*Background and Objectives:* This study aimed to evaluate the effectiveness of immunotherapy compared to chemotherapy across different treatment lines in patients with pleural mesothelioma. It also sought to identify factors influencing the success of immunotherapy, such as histological subtype, PD-L1 expression, type of asbestos exposure, and metastatic status. *Materials and Methods:* A retrospective analysis was conducted with 60 patients diagnosed with pleural mesothelioma. Data on age, gender, histological subtype, and asbestos exposure were collected for all patients and PD-L1 expression was assessed in a subset of patients. Patients received either chemotherapy or immunotherapy as first-, second-, and third-line treatments, and progression-free survival (PFS) and treatment responses were evaluated. *Results:* Among the 60 patients, 35 (58.3%) were male and the median age was 59 years. The majority (71.7%) had epithelioid histology and 28.3% had distant metastases. Asbestos exposure was documented in 65% of the cases. PD-L1 expression of ≥1% was found in 13 of 17 patients tested. First-line treatments included immunotherapy for 11 patients and chemotherapy for the others, with immunotherapy achieving median PFS of 9 months versus 6 months for chemotherapy, although the difference was not statistically significant. In third-line treatments, immunotherapy significantly outperformed chemotherapy with median PFS of 6 months compared to 3 months (*p* = 0.048). Absence of metastasis and prior asbestos exposure in an endemic region were linked to better immunotherapy outcomes. *Conclusion:* Immunotherapy shows increased efficacy in later treatment lines for pleural mesothelioma, especially for patients without metastases or with prior endemic asbestos exposure. Tailored therapeutic strategies should be further explored in prospective studies.

## 1. Introduction

Pleural mesothelioma (PM) is a highly aggressive disease with a median survival period of approximately 1 year, while the 5-year survival rate for PM is approximately 10% [1,2]. The aetiological cause is asbestos exposure, with the disease typically manifesting years after the initial exposure [3]. Age, gender, disease stage, and histological subtype are prognostic factors in PM [4,5]. Despite the limited efficacy of chemotherapy in treating PM, the combination of platinum-based chemotherapy and pemetrexed is a recommended treatment option [6]. However, the addition of bevacizumab to the treatment regimen has also been observed to confer a survival advantage [7]. The efficacy of cytotoxic therapies in the treatment of advanced pleural mesothelioma is limited, highlighting the need for new treatment options. Monoimmunotherapy and its combinations show promising results. The efficacy of combinations of immunotherapy has been demonstrated in the treatment of PM, particularly for the sarcomatoid subtype, as evidenced by the findings of the CheckMate 743 study [8]. Given the association between asbestos exposure and chronic inflammation, there are insufficient data on the efficacy of immunotherapy in these patients.

## 2. Materials and Methods

This research was conducted as a Turkish Oncology Group (TOG) study, designed as a retrospective observational multicentre study. Eight centres in Türkiye took part in this study. The study population comprised 60 patients aged 18 years or above with metastatic or unresectable PM who had received immunotherapy at any stage of treatment between January 2014 and January 2024. Patients who had not undergone immunotherapy for the treatment of mesothelioma or who were in the early stages of the disease were excluded from this study. The demographic, clinical, and laboratory data of the patients were retrospectively obtained from hospital archive systems. The following variables were recorded: gender, age at diagnosis, stage of disease at presentation, Eastern Cooperative Oncology Group performance status (ECOG PS), histological subtype, type of surgical intervention, and radiotherapy, immunotherapy, and chemotherapy treatments. The disease stage was determined in accordance with the 8th edition guidelines of the American Joint Committee on Cancer (AJCC). Treatment response was evaluated at 3-month intervals using thoracic and abdominal tomography or positron emission tomography and assessed according to the Response Evaluation Criteria in Solid Tumours v1.1 (RECIST). The objective of this study was to investigate the treatments received by patients in initial, subsequent, and additional treatment lines, as well as their response status, overall survival (OS), and progression-free survival (PFS). PFS durations obtained with immunotherapy from any line of treatment and parameters that could have potential effects on PFS were evaluated using univariate and multivariate analyses. Specifically, the effects of the following variables on PFS were analysed: age, gender, smoking status, histological subtype, programmed death ligand 1 (PD-L1) level [PD-L1 expression was measured using the immunohistochemical method, VENTANA PD-L1 (SP263)], type of asbestos exposure, radiotherapy status, presence of distant metastasis, immunotherapy type (monotherapy versus combination immunotherapy), immunotherapy lines, and steroid use during treatment. The treatment regimens given to patients are nivolumab (3 mg/kg, IV, once every two weeks), ipilimumab (1 mg/kg, IV, once every six weeks), pembrolizumab (200 mg, IV, once every 3 weeks), and bevacizumab (15 mg/kg, IV, once every 3 weeks combined with platinum-based chemotherapy).

Statistical analysis of the data obtained in this study was conducted using PASW Statistics for Windows, Version 18.0 (SPSS Inc., Chicago, IL, USA). Descriptive statistics were employed to evaluate patient characteristics and parameter frequency, while the chi-square test and Fisher’s exact test were utilised to assess relationships between parameters. Kaplan–Meier survival analysis was employed for survival analyses with the log-rank *p*-value serving as the basis for interpretation. Cox regression analysis was utilised for univariate and multivariate survival analyses, with the enter method employed for univariate analysis and the backward stepwise likelihood ratio method utilised for multivariate analysis. Confidence intervals of 95% and *p*-values of less than 0.05 were accepted as indicators of statistical significance.

## 3. Results

A total of 60 patients were included in this study, comprising 35 men (58.3%) and 25 women (41.7%). The median age at diagnosis was 59 years (range: 34–83 years). Forty-three (71.7%) of the patients exhibited epithelioid histology. Distant metastasis was identified in 17 patients (28.3%) and a total of 12 patients (20%) required steroid use due to adverse effects or other reasons. The PD-L1 level was analysed for 17 (28.3%) patients, with a PD-L1 level of ≥1% observed in 13 (21.7%) patients. A known history of asbestos exposure was documented in 39 (65%) cases, with the remainder of the patients having an unknown mode of exposure. Further findings are summarised in Table 1.

Analysis of the treatments received by the patients revealed that all patients had undergone at least one line of treatment, 57 patients had received two lines of treatment, and 32 patients had undergone at least three lines of treatment. Immunotherapy was employed for 11 (18.3%) patients in the first line of treatment, 27 (47.4%) patients in the second line, and 22 (36.7%) patients in subsequent stages. Among all steps of treatment, 7 patients (11.7%) received combination immunotherapy, and 53 patients (88.3%) received immunotherapy in the form of monotherapy (Table 2).

**Figure 1:**
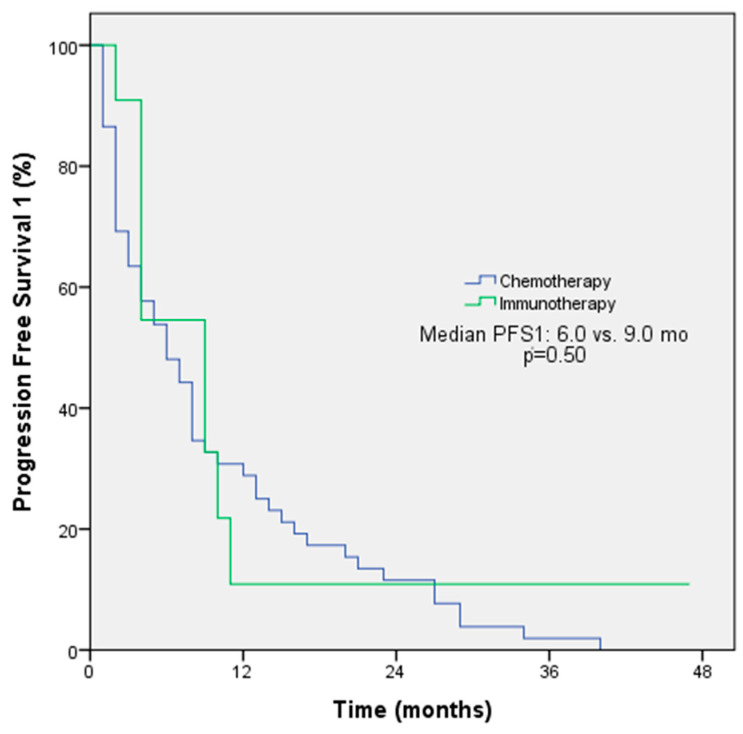
PFS1 Kaplan–Meier survival analysis according to treatment regimens.

**Figure 2:**
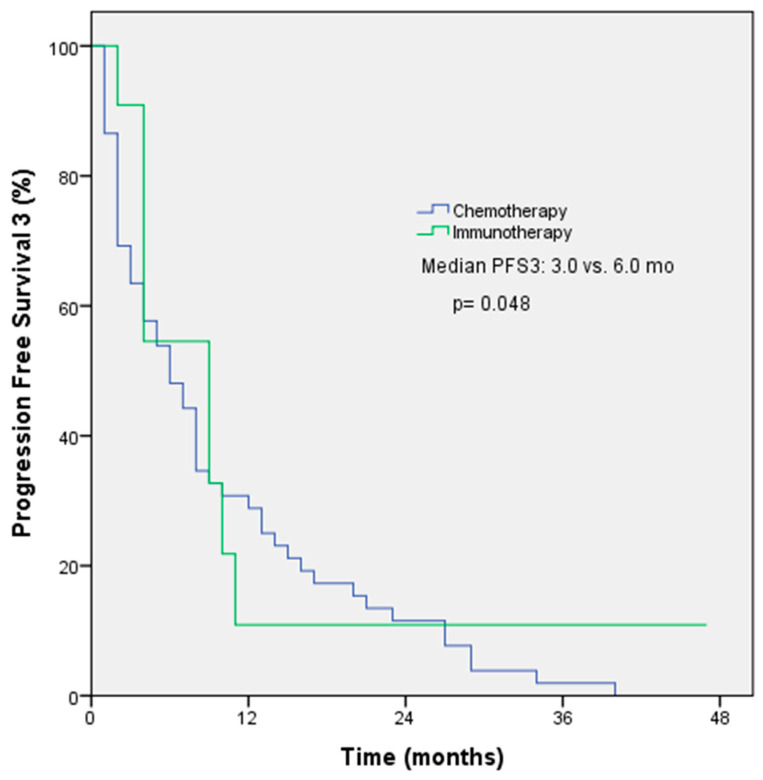
PFS3 Kaplan–Meier survival analysis according to treatment regimens.

The objective response rate (ORR) was 54.5% among patients receiving immunotherapy and 55.1% among patients receiving chemotherapy in first-line treatment (*p* = 0.97). The median PFS for first-line treatment was 9 months with immunotherapy and 6 months with chemotherapy (*p* = 0.50, Figure 1). The ORRs were 33.3% among patients receiving immunotherapy and 30% among patients receiving chemotherapy in second-line treatment (*p* = 0.78); the median PFS for second-line treatment was 3 months with immunotherapy and 6 months with chemotherapy (*p* = 0.30). The ORRs for subsequent lines of treatment with immunotherapy and chemotherapy were comparable at 47.6% and 30%, respectively. However, the median PFS was longer with immunotherapy (6 months) compared to chemotherapy (3 months). The *p*-value for this difference was 0.048, indicating a statistically significant difference (Figure 2). The ORR was 57.1% and the PFS was 4 months with immunotherapy in the form of monotherapy, while the ORR was 37.7% (*p* = 0.42) and the PFS was 4 months (*p* = 0.44) with combination therapy in any line of treatment (Table 3).

In univariate analysis, no significant relationship was identified between the variables of age, gender, ECOG PS, smoking, histological subtype, PD-L1 level, asbestos exposure type, radiotherapy status, presence of distant metastasis, immunotherapy type, line of immunotherapy use, or steroid use during treatment and PFS. However, in multivariate analysis, asbestos exposure type and presence of distant metastasis were found to be associated with immunotherapy efficacy. The absence of distant metastasis [hazard ratio (HR): 4.59; 95% confidence interval (CI): 1.02–20.7; *p* = 0.04] and asbestos exposure resulting from residence in an endemic area (HR: 4.16; 95% CI: 1.04–16.7; *p* = 0.04) were identified as positive predictive parameters for PFS achieved with immunotherapy (Table 4). The ORR and PFS values of the parameters evaluated in the multivariate analysis are detailed in Table 5.

No grade 4 side effects related to immunotherapy were observed. Grade 3 pneumonitis was observed in three (5.1%) patients.

## 4. Discussion

Although PM is a rare disease, it represents a significant public health concern in regions where it is endemic, largely due to its aggressive nature. As is the case with many cancers, the efficacy of chemotherapy in PM has primarily been investigated in the early stages of its development to date. In a phase III study conducted by Vogelzang et al., in 2003 [6], the contribution of pemetrexed to cisplatin in the first-line treatment of patients with unresectable pleural mesothelioma was investigated. The addition of pemetrexed to cisplatin was observed to result in a 3.3-month OS benefit. The MAPS study was designed to investigate the contribution of adding bevacizumab to pemetrexed + cisplatin in first-line treatment. In this study, a 2.1-month contribution to PFS was observed in the bevacizumab arm, and OS was reported as 16.1 months in the pemetrexed + cisplatin arm and 18.8 months in the pemetrexed + cisplatin + bevacizumab arm (HR: 0.77; *p* = 0.0167) [7]. In our study, 81.7% of patients received chemotherapy as their first-line treatment. The majority of patients received platinum-based combination therapy, while 25% received bevacizumab. The median OS was 21 months and the median PFS was 6 months in the first-line chemotherapy group. These values are similar to those reported in the literature for first-line chemotherapy combinations.

Second-line and subsequent treatments of PM may be conducted with gemcitabine, platinum therapies, and vinorelbine [9]. In a phase II study, an ORR of 24% was obtained with vinorelbine [10]. In the RAMES study, which compared gemcitabine plus ramucirumab with single-agent gemcitabine in patients who had progressed after pemetrexed + platinum, the median OS was 13.8 months in the gemcitabine + ramucirumab arm and 7.5 months in the gemcitabine arm (HR: 0.71) [11]. In our study, 52.6% of patients received chemotherapy in the second line of treatment. There was considerable heterogeneity in terms of the chemotherapy protocols used. However, the overall ORR was 30% and median PFS was 6 months with second-line chemotherapy in our study.

The use of immunotherapy has recently gained prominence in the treatment of numerous cancer types, particularly for cases of melanoma, renal cell carcinoma, and lung cancers [12,13,14,15,16,17,18]. The utilisation of immunotherapy in the context of PM has commenced primarily as a salvage treatment for patients who have experienced disease progression in conjunction with chemotherapy. In the phase IIb DETERMINE study, tremelimumab was compared with a placebo in patients with pleural and peritoneal mesothelioma who had received one or two prior lines of treatment. No significant difference in OS was observed between the groups (HR: 0.92, *p* = 0.41) [19]. In the PROMISE study, pembrolizumab was compared with physician-selected chemotherapy (gemcitabine or vinorelbine) in second-line treatment. The median PFS for patients receiving pembrolizumab was 2.5 months compared to 3.4 months for those receiving chemotherapy (HR: 1.06, *p* = 0.76), while the median OS for patients receiving pembrolizumab was 10.7 months compared to 12.4 months for those receiving chemotherapy (HR: 1.12, *p* = 0.59). Thus, no statistically significant difference was observed between the two groups in terms of PFS or OS [20]. The phase III CONFIRM study included patients with PM who had received more than one line of treatment. In that study, the efficacy of nivolumab was compared to that of a placebo. The median PFS was 3 months in the nivolumab arm and 1.8 months in the placebo arm (HR: 0.67, *p* = 0.0012), while the median OS was 10.2 months in the nivolumab arm and 6.9 months in the placebo arm (HR: 0.69, *p* = 0.009). The study demonstrated that nivolumab conferred superior PFS and OS compared to a placebo in the treatment of PM irrespective of histology [21]. In the phase II MAPS2 study, the efficacy rates of nivolumab + ipilimumab combination therapy and nivolumab monotherapy were evaluated in patients with unresectable PM who had received one or two lines of treatment. The ORR was 28% and 19% in the nivolumab + ipilimumab arm and the nivolumab arm, respectively. The median PFS was 5.6 months and 4 months, respectively, while median OS was 15.9 months and 11.9 months [4]. In our study, as second-line treatment, 3 patients (5.3%) received nivolumab + ipilimumab combination treatment, while 24 patients (42.1%) received nivolumab monotherapy. The ORR was 30% in the chemotherapy arm and 33.3% in the immunotherapy arm, with a *p*-value of 0.78. For second-line treatment, the median PFS was 6 months in the chemotherapy arm and 3 months in the immunotherapy arm, with a *p*-value of 0.30. In treatment phases following the second line, 1 (3.1%) patient received nivolumab + ipilimumab, while 21 (65.6%) patients received nivolumab monotherapy. The ORR was 30% in the chemotherapy arm and 47.6% in the immunotherapy arm (*p* = 0.35), while the median PFS was 3 months in the chemotherapy arm and 6 months in the immunotherapy arm (*p* = 0.048).

The results of our study indicate that the use of immunotherapy in second-line and subsequent treatment arms yielded outcomes comparable to those of chemotherapy with respect to ORR. Although no significant difference in PFS was observed between immunotherapy and chemotherapy in second-line treatment, immunotherapy resulted in superior PFS in subsequent treatment lines. This may be attributed to the limited efficacy of the treatment options employed following second-line treatment.

The most important study conducted to date on the utilisation of immunotherapy in the initial treatment of PM is the CheckMate 743 study [8]. In this multicentre randomised phase III study, the efficacy of pemetrexed + platinum treatment, representing the standard of care, was compared to that of a nivolumab + ipilimumab immunotherapy combination in patients with advanced-stage PM who had not received prior treatment. In this study, which was stratified according to histology, the median OS was 14.1 months in the chemotherapy arm and 18.1 months in the immunotherapy arm (HR: 0.74, *p* = 0.0020). While no significant difference was observed in median OS values between the two arms for epithelioid histology (16.5 months versus 18.7 months; HR: 0.86), median OS for non-epithelioid histology was 8.8 months in the chemotherapy arm and 18.1 months in the immunotherapy arm (HR: 0.46). Thus, the results of this study indicate that the utilised immunotherapy combination is effective in the treatment of both histologies, with particularly notable efficacy in cases of sarcomatoid histology, where chemotherapy efficacy is low. The number of patients receiving first-line immunotherapy in our study was limited. Nevertheless, in our study, the ORR was 54.5%, median PFS was 9 months, and median OS was 15 months with first-line immunotherapy treatment. The efficacy of immunotherapy and chemotherapy in first-line treatment in our study was found to be comparable to the previous results in the literature. The analysis of the results of combination or mono-immunotherapy used in any line of this study revealed an ORR of 57.1%, median PFS of 4 months, and median OS of 31 months with combination therapy. In the monotherapy arm, the ORR was 37.7%, median PFS was 4 months, and median OS was 18 months. No statistically significant differences were observed in ORR, PFS, or OS between the two groups. There was a numerical difference in median OS between the two groups, which we think was due to the small number of patients in the combination arm. This lack of a difference between immunotherapy and chemotherapy responses in first-line treatment in our study may be attributed to a variety of factors. In the course of our study, some patients underwent neoadjuvant or adjuvant treatments. Additionally, 25% of the patients in this study received bevacizumab in conjunction with chemotherapy. In contrast, the control arm of the CheckMate 743 study did not include bevacizumab as a component. Furthermore, the majority of patients in this study received monotherapy as the first-line treatment.

Despite the absence of a robust biomarker that accurately predicts immunotherapy response, evidence suggests a strong correlation between immunotherapy treatment response and PD-L1 levels in certain cancer types, particularly lung cancers [22,23]. In the subgroup analysis of the CheckMate 743 study, it was demonstrated that PD-L1 positivity and, in particular, sarcomatoid histology may predict immunotherapy response in comparison to chemotherapy [8]. In the present study, we evaluated 11 parameters that may be related to immunotherapy response, including age, gender, histology, smoking status, type of asbestos exposure, history of radiotherapy, PD-L1 status, presence of distant metastasis, treatment line in which immunotherapy was used, use of monotherapy versus combination immunotherapy, and steroid use due to side effects or for other reasons. In the multivariate analysis of our study, it was found that living in a region with endemic asbestos and the absence of distant metastasis were predictive parameters for PFS. The better response to immunotherapy of patients without distant metastases may be due to the fact that local disease develops in a chronic inflammatory environment and immunotherapy plays a more effective role in that environment. In some regions of Türkiye, including Eskisehir, Kutahya, Bilecik, Yozgat, Sivas, and Diyarbakir, asbestos is found within the natural soil pattern. This substance, called ‘white soil’, has been used in socio-cultural contexts as a heat and water insulator and in the plaster and roof insulation of houses. Therefore, people living in these regions have been chronically exposed to asbestos in the natural course of life [24,25,26]. This ‘white soil’, which is commonly used in Türkiye, contains tremolite asbestos fibres and is often contaminated with chrysotile-type fibres [25,26,27]. The biology of PM shows significant heterogeneity in both tumours and the microenvironment. A major role of chronic inflammation and local tumour suppression in tumorigenesis has been observed in some experimental models as well as spontaneous regressions attributable to an activation of the immune system [28]. In our study, we found that the efficacy of immunotherapy was better in patients with a history of chronic exposure to asbestos fibres, as reported above, who primarily lived in the endemic region of Diyarbakir. This suggests that exposure to different types of asbestos in the process of mesothelioma formation triggers the process leading to carcinogenesis by different mechanisms and creates different microenvironments for the disease. These findings are not aligned with those of the CheckMate 743 study. One potential explanation for this discrepancy is the relatively low number of patients with known PD-L1 levels in our study.

The limitations of our study include its retrospective nature, lack of a control arm, the use of immunotherapy at different stages of treatment, the heterogeneity of treatment options, the use of different chemotherapy options, and the relatively small number of patients. In addition, genetic factors (BAP-1, etc.) were not analysed in our study and their effect on immunotherapy responses was not evaluated due to the retrospective nature of this study.

## 5. Conclusions

In conclusion, our findings indicate that immunotherapy achieves ORR, PFS, and OS values comparable to those observed with chemotherapy. Notably, patients with endemic asbestos exposure and the absence of distant metastasis may demonstrate enhanced responsiveness to immunotherapy.

## Figures and Tables

**Table 1:** General characteristics of the patients.

	*n* = 60 (%)
**Age, years, median**	59 (34–83)
**Gender**	
Male	35 (58.3)
Female	25 (41.7)
**ECOG PS**	
0	18 (30)
1–2	42 (70)
**Smoking**	
Yes	22 (36.7)
No	37 (61.7)
Unknown	1 (1.6)
**Histopathological type**	
Epithelioid	43 (71.7)
Sarcomatoid or biphasic	12 (20)
Others	5 (8.3)
**Distant metastasis**	
Yes	17 (28.3)
No	43 (71.7)
**Perioperative Therapy**	
Yes	24 (40)
No	36 (60)
**Previous radiation therapy**	
Yes	29 (48.3)
No	31 (51.7)
**Type of surgery (n = 24)**	
EPP	7 (29.2)
PD	16 (66.7)
Others	1 (4.1)
**Steroid use**	
No	48 (80)
Yes	12 (20)
**Asbestos exposure**	
Living in an endemic region	39 (65)
Other	21 (35)
**PD-L1 status**	
Unknown	43 (71.7)
≥1%	13 (21.7)
<1%	4 (6.7)

ECOG PS: Eastern Cooperative Oncology Group performance status; PD-L1: programmed death ligand 1; EPP: extrapleural pneumonectomy; PD: pleurectomy decortication.

**Table 2:** Treatments received by the patients.

	n (%)
**First-line treatment options (n = 60)**	
Nivolumab + ipilimumab	3 (5)
Nivolumab	6 (10)
Platinum-based Ch + beva	15 (25)
Platinum-based Ch	27 (45)
Other Ch	7 (11.7)
Pembrolizumab	2 (3.3)
**Second-line treatment options (n = 57)**	
Nivolumab + ipilimumab	3 (5.3)
Nivolumab	24 (42.1)
Platinum-based Ch + beva	4 (7)
Platinum-based Ch	18 (31.6)
Other Ch	8 (14)
**Subsequent treatment options (n = 32)**	
Nivolumab + ipilimumab	1(3.1)
Nivolumab	21 (65.6)
Chemotherapy	10 (31.3)
**Immunotherapy, any line (n = 60)**	
Nivolumab + ipilimumab	7 (11.7)
Nivolumab	51 (85)
Pembrolizumab	2 (3.3)

Ch: chemotherapy; beva: bevacizumab.

**Table 3:** Evaluation of treatment responses.

	n (%)	ORR %	*p*	mPFS (mo)	*p*	mOS (mo)	*p*
**First-line treatment options (n = 60)**			0.97 *		0.50 **		0.49 **
Immunotherapy	11 (18.3)	6 (54.5)		9		15	
Chemotherapy	49 (81.7)	27 (55.1)		6		21	
**Second-line treatment options (n = 57)**			0.78 *		0.30 **		
Immunotherapy	27 (47.4)	9 (33.3)		3			
Chemotherapy	30 (52.6)	9 (30)		6			
**Subsequent treatment options (n = 32)**			0.35 *		0.048 **		
Immunotherapy	22 (68.8)	10 (47.6)		6			
Chemotherapy	10 (31.2)	3 (30)		3			
**Immunotherapy, any line (n = 60)**			0.42 ***		0.44 **		0.64 **
Nivolumab + ipilimumab	7 (11.7)	4 (57.1)		4		31	
Nivolumab or pembrolizumab	53 (88.3)	20 (37.7)		4		18	

*: Pearson chi-square; **: log-rank *p*-value; ***: Fisher’s exact test; ORR: objective response rate; mPFS: median progression-free survival; mOS: median overall survival; mo: months.

**Table 4:** Results of univariate and multivariate analyses of parameters potentially influencing progression-free survival with immunotherapy.

		Univariate Analysis			Multivariate Analysis	
	HR	95% CI	*p*	HR	95% CI	*p*
Age, years (<58 */≥58)	0.60	0.34–1.07	0.08			
Gender (female */male)	0.89	0.50–1.58	0.70			
ECOG PS (0 */1–2)	1.36	0.75–2.47	0.36			
Smoking (no */yes)	0.94	0.52–1.72	0.85			
Histological subtypes (epithelioid */others)	0.88	0.46–1.67	0.71			
Asbestos exposure (endemic region */others)	1.10	0.60–2.01	0.74	4.16	1.04–16.7	0.04
Previous radiation therapy (no */yes)	1.10	0.62–1.94	0.74			
PD-L1 (<1% */≥1%)	0.98	0.25–3.83	0.98			
Distant metastasis (no */yes)	1.37	0.73–2.57	0.32	4.59	1.02–20.7	0.04
Treatment line (first */subsequent)	1.38	0.67–2.88	0.37			
Treatment option (mono IO */combination IO)	0.72	0.28–1.82	0.48			
Steroid use (no */yes)	0.95	0.47–1.93	0.89			

*: reference category; HR: hazard ratio; CI: confidence interval; ECOG PS: Eastern Cooperative Oncology Group performance status; PD-L1: programmed death ligand 1; IO: immunotherapy.

**Table 5:** Response rates and PFS durations according to subgroups.

	n	ORR %	*p*	mPFS (mo)	*p* (log-Rank)
**Age (years)**			0.20 *		0.06
<58	26	8 (30.8)		3	
≥58	34	16 (47.1)		5	
**Gender**			0.59 *		0.67
Female	25	9 (36)		4	
Male	35	15 (42.9)		4	
**Smoking**			0.66 *		0.84
No	37	16 (42.1)		4	
Yes	22	8 (36.4)		4	
**Histological subtypes**			0.55 *		0.68
Epithelioid	43	18 (41.9)		4	
Others	17	6 (35.3)		3	
**Asbestos exposure**			0.43 *		0.62
Endemic region	39	17 (43.6)		4	
Others	21	7 (33.3)		4	
**Previous radiation therapy**			0.68 *		0.73
No	31	12 (38.7)		3	
Yes	29	12 (41.4)		4	
**PD-L1**			0.58 **		0.98
<1%	4	2 (50.0)		4	
≥1%	13	9 (69.2)		6	
**Distant metastasis**			0.48 *		0.26
No	43	16 (37.2)		4	
Yes	17	8 (47.1)		3	
**Treatment line**			0.32 **		0.32
First line	11	6 (54.5)		6	
Subsequent	49	18 (36.7)		3	
**Treatment option**			0.42 **		0.44
Mono IO	53	20 (37.7)		4	
Combination IO	7	4 (57.1)		4	
**Steroid use**			0.74 *		0.88
No	48	20 (41.7)		4	
Yes	12	4 (33.3)		4	

*: Pearson chi-square; **: Fisher’s exact test; ORR: objective response rate; mPFS: median progression-free survival; PD-L1: programmed death ligand 1; IO: immunotherapy.

## Data Availability

Data are available upon request from the corresponding author.
