# Correction: Kalkan et al. Efficacy of Immunotherapy Versus Chemotherapy in Advanced Pleural Mesothelioma: A Turkish Oncology Group (TOG) Study. *Medicina* 2025, *61*, 638

**DOI:** 10.3390/medicina61111997

**Published:** 2025-11-07

**Authors:** Ziya Kalkan, Senar Ebinc, Murat Arcagok, Ahmet Bilici, Ozcan Yildiz, Saadettin Kilickap, Deniz Can Guven, Ali Murat Tatli, Ahmet Taner Sumbul, Nil Molinas Mandel, Akin Ozturk, Murat Bardakci, Serdar Karakaya, Muhammet Ali Kaplan

**Affiliations:** 1 Department of Medical Oncology, Mardin Training and Research Hospital, Mardin 47100, Türkiye; 2 Department of Medical Oncology, Van Yuzuncu Yil University Faculty of Medicine, Van 65100, Türkiye; senarebinc@gmail.com; 3 Department of Medical Oncology, Dicle University Faculty of Medicine, Diyarbakir 21100, Türkiye; judge_murat@hotmail.com (M.A.); drmalikaplan@hotmail.com (M.A.K.); 4 Department of Medical Oncology, Faculty of Medicine, Medipol University, Istanbul 34000, Türkiye; ahmetknower@yahoo.com (A.B.); oyildiz@medipol.edu.tr (O.Y.); 5 Department of Medical Oncology, Istinye University Faculty of Medicine, Istanbul 34000, Türkiye; skilickap@yahoo.com; 6 Department of Medical Oncology, Hacettepe University Cancer Institute, Ankara 06000, Türkiye; denizcguven@hotmail.com; 7 Department of Medical Oncology, Akdeniz University Faculty of Medicine, Antalya 07000, Türkiye; alimurattat@hotmail.com; 8 Department of Medical Oncology, Baskent University Adana Dr. Turgut Noyan Application and Research Center, Adana 01100, Türkiye; asumbul3188@gmail.com; 9 Department of Medical Oncology, American Hospital, Istanbul 34000, Türkiye; nmmandel@gmail.com; 10 Department of Medical Oncology, Sureyyapasa Chest Diseases and Chest Surgery Training and Research Hospital, Istanbul 34000, Türkiye; onkoakin@gmail.com; 11 Department of Medical Oncology, Gazi Yasargil Training and Research Hospital, Diyarbakir 21100, Türkiye; dr.muratbardakci@hotmail.com; 12 Department of Medical Oncology, Ankara Ataturk Sanatoryum Training and Research Hospital, Ankara 06000, Türkiye; drserdarkarakaya@gmail.com

## Error in Figure

In the original publication, there was a mistake in Figure 2 as published [1]. Only the Kaplan–Meier curve needs to be updated because it is currently identical to the one in Figure 1. The corrected Figure 2 is as follows. The authors state that the scientific conclusions are unaffected. This correction was approved by the Academic Editor. The original publication has also been updated.

## Figures and Tables

**Figure 2:**
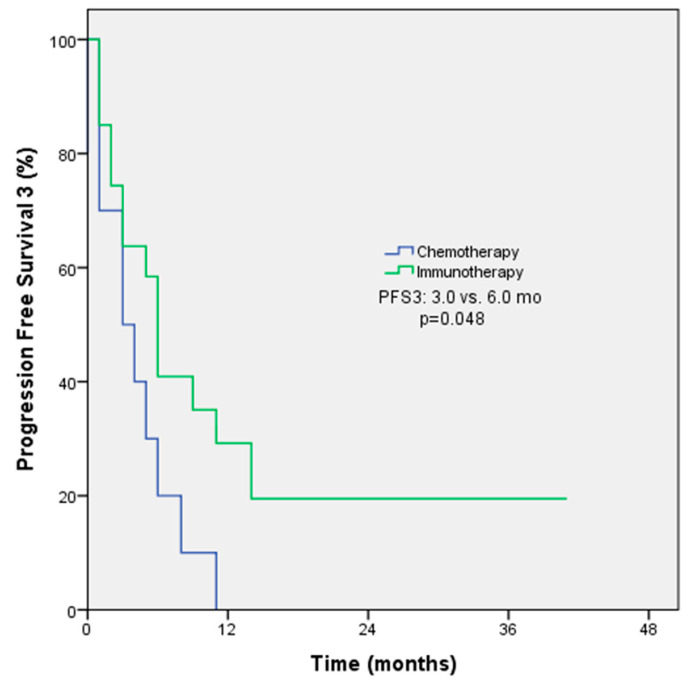
PFS3 Kaplan–Meier survival analysis according to treatment regimens.
